# A Narrative Review of Quercetin’s Role as a Bioactive Compound in Female Reproductive Disorders

**DOI:** 10.3390/nu17071118

**Published:** 2025-03-24

**Authors:** Yasaman Khamineh, Mahsa Ghiasvand, Sanaz Panahi-Alanagh, Parisa Rastegarmand, Samaneh Zolghadri, Agata Stanek

**Affiliations:** 1 Department of Animal Sciences and Marine Biology, Faculty of Life Sciences and Biotechnology, Shahid Beheshti University, Tehran 1983969411, Iran; yasaman.khamine@gmail.com (Y.K.); mahsaghiasvandd1998m@gmail.com (M.G.); 2 Department of Stem Cells and Developmental Biology, Cell Science Research Centre, Royan Institute for Stem Cell Biology and Technology, Academic Center for Education, Culture and Research (ACECR), Tehran 3365166571, Iran; 3 Department of Plant and Animal Biology, Faculty of Biological Science and Technology, University of Isfahan, Isfahan 817467344, Iran; sanazpanahialanagh@gmail.com; 4 Department of Microbiology, College of Science, Shiraz Branch, Islamic Azad University, Shiraz 1477893780, Iran; p.rastegar1991@gmail.com; 5 Department of Biology, Jahrom Branch, Islamic Azad University, Jahrom 7414785318, Iran; 6 Department of Internal Medicine, Metabolic Diseases and Angiology, Faculty of Health Sciences in Katowice, Medical University of Silesia, Ziolowa 45/47 St., 40-635 Katowice, Poland

**Keywords:** female reproductive disorders, quercetin, polycystic ovary syndrome, gynecological cancers, pregnancy loss

## Abstract

Lifestyle, nutrition, and metabolic status are central to maintaining women’s reproductive health. With the rising prevalence of infertility, the need for effective strategies to preserve and enhance women’s reproductive well-being has become increasingly urgent. Quercetin, a plant-derived polyphenol, has attracted growing interest for its potential to support reproductive health, regulate the hormonal balance, and improve fertility. This narrative review examines Quercetin’s role in women’s reproductive health and delineates its possible mechanisms of action in female reproductive system disorders, including polycystic ovary syndrome, recurrent miscarriage, and cervical, ovarian, and endometrial cancer (EC). By highlighting the biological pathways through which quercetin may exert its effects, this article underscores the need for further research and clinical trials to validate its therapeutic potential and its applications as a bioactive compound in foods aimed at enhancing women’s reproductive health.

## 1. Introduction

Concerns regarding female fertility and long-term reproductive health have escalated in response to the increasing prevalence of conditions such as polycystic ovary syndrome (PCOS) [1], various gynecological cancers [2], and pregnancy challenges such as recurrent miscarriages [3]. A substantial body of epidemiological and clinical evidence suggests that plant-based diets can reduce the risk of chronic diseases, including cancer [4,5]. Bioactive compounds derived from natural sources have garnered attention for their potential to address these multifactorial challenges. Among these bioactive compounds, Quercetin [2-(3,4-dihydroxyphenyl)-3,5,7-trihydroxy-4-Hchromen-4-one] is a widely consumed dietary flavonoid present in a variety of foods, including fruits, green leafy vegetables, seeds, buckwheat, nuts, flowers, barks, broccoli, olive oil, apples, onions, green tea, red grapes, red wine, dark cherries, and berries like blueberries and cranberries [6]. In addition, numerous studies have shown that Quercetin, when taken in acceptable amounts, may provide beneficial biological effects, including antioxidant, anticancer, and anti-inflammatory properties [7]. By scavenging reactive oxygen species (ROS) and reducing inflammation, Quercetin could enhance the health of reproductive tissues and lower the risk of related conditions [8]. Moreover, Quercetin has shown promise in balancing hormones and reducing excess androgens [9]. Additionally, by mitigating inflammation and potentially improving how the body uses insulin, Quercetin could help regularize women’s periods [10].

Quercetin also has the potential to affect various pathological processes associated through multiple targets and pathways [11], making it a promising candidate for addressing these health issues. Quercetin decreases apoptosis by blocking Akt/PKB phosphorylation, minimizing mitochondrial cyst release, and downregulating Cu–Zn peroxidase dismutase to suppress the ROS reaction [12]. Similarly, it was observed that Quercetin protects primordial follicles in mice by inhibiting the activation of the PI3K/Akt/FOXO3a pathway [13]. Recently, researchers are boosting Quercetin’s efficacy through encapsulation, combining it with antioxidants, or selectively breeding specific fruits and vegetables offers the potential to naturally increase the Quercetin content, allowing for the development of tailored, Quercetin-rich foods for specific health benefits [9,10,14].

Despite the existing studies on the benefits of quercetin, there remains a significant gap in the literature regarding a comprehensive review specifically focused on its effects on the female reproductive system. In this narrative review, we will explore a diverse array of research on Quercetin, a bioactive compound derived from plants, and its effects on female reproductive health issues, including PCOS, pregnancy loss, and gynecological cancers. Additionally, we will examine the potential of Quercetin-based functional foods as an innovative and accessible strategy to enhance women’s reproductive health. Our review will include an analysis of in vitro/vivo studies, and clinical trial research, along with insights into the origin, metabolism, and pharmacokinetics properties of Quercetin.

## 2. Methods

This narrative review conducted a literature search across four principal scientific databases: PubMed, ScienceDirect, Google Scholar, and Web of Science, focusing on relevant studies up to 2025. The search strategy utilized to identify applicable articles was as follows: ((((“plant-based diet”) OR (“polyphenol”)) OR (“quercetin-based foods”)) OR (“quercetin”)) AND ((((((((((((“polycystic ovary syndrome”) OR (“PCOS”)) OR (“pregnancy”)) OR (“pregnancy loss”)) OR (“miscarriage”)) OR (“gynecological cancer”)) OR (“cervical cancer”)) OR (“ovarian cancer”)) OR (“endometrial cancer”)) OR (“anticancer”)) OR (“antioxidant”)) OR (“anti-inflammatory”)). All abstracts generated from this search were screened to pinpoint relevant studies. Moreover, a manual search was performed to answer secondary research questions. The inclusion criteria encompassed original research articles, in vitro and in vivo studies, clinical trials, and narrative or systematic reviews that investigated quercetin’s mechanisms of action and its therapeutic potential in female reproductive health. Studies were excluded if they did not correlate with reproductive function, failed to examine mechanistic pathways, or centered on compounds unrelated to quercetin.

## 3. Effect of Quercetin on Pregnancy and Miscarriage

Recent multinational research has indicated a growing prevalence of herbal medicine use during pregnancy [15]. However, this trend raises concerns, as many women lack sufficient awareness of the phytochemical properties of these products, leading to improper usage, and, consequently, they may inadvertently jeopardize their health and that of their unborn children [16]. Spontaneous pregnancy loss prior to viability, commonly referred to as miscarriage, represents a significant challenge in reproductive health. The absence of a universally standardized definition for this condition complicates the precise assessment of its prevalence, with reported rates varying between 1% and 5% [17]. Recurrent pregnancy loss (RPL), defined as the occurrence of multiple consecutive miscarriages, is estimated to affect 2% to 5% of couples, often resulting in profound emotional distress and disrupting family planning endeavors. Etiological factors associated with RPL include chromosomal anomalies, defective placentation, thrombotic disorders, and unfavorable uterine conditions [18,19,20]. Among these, oxidative stress has been identified as a critical factor in early pregnancy failure. Evidence suggests that premature and disorganized maternal blood flow to the placenta is associated with elevated oxidative damage in placental tissues, further contributing to pregnancy loss [21], and disruptions in the equilibrium between oxidative stress and inflammatory processes have been implicated in the pathogenesis of these pregnancy-related immune disorders [22]. Oxidative stress (OS) has been shown to impair placental function and disrupt fetal development through multiple mechanisms. These include the dysregulation of critical nutrient transporters, such as Slc2a1 and Slc38a1, as well as the induction of cellular apoptosis [23,24,25]. Such disruptions can lead to severe pregnancy complications, including embryonic resorption, recurrent pregnancy loss, intrauterine growth restriction (IUGR), and fetal demise [26]. In women with recurrent abortions, the increased generation of radical species by leukocytes was demonstrated via increased granulocyte spontaneous chemiluminescence [27]. Studies have shown that Quercetin plays a vital role in reducing oxidative stress during trophoblast syncytialization, a critical process in placental development [28]. Disruptions in syncytialization, often caused by oxidative stress and mitochondrial dysfunction, are implicated in pregnancy-related complications, including hypertensive disorders of pregnancy (HDP). In HDP-affected placentas, mitochondrial dysfunction results in reactive oxygen species (ROS) overproduction, which further impairs syncytialization [29,30]. Recent studies have demonstrated that Quercetin treatment (5 µM) for 48 h facilitates trophoblast fusion by upregulating syncytialization markers and enhancing mitochondrial fusion and mitophagy, as evidenced by immunofluorescence analysis. Although cell fusion inherently generates ROS, Quercetin effectively counteracts oxidative stress by regulating key antioxidant pathways, thereby supporting placental health and function [28]. In vitro studies conducted by Wang et al. revealed that 1 μM, 5 μM, and 10 μM of Quercetin have the potential to reduce the incidence of spontaneous abortion (SA) by modulating inflammatory responses and apoptotic pathways in HTR-8/SVneo cells exposed to lipopolysaccharide (LPS). Inflammatory responses, especially those triggered by LPS-induced immune activation, are widely acknowledged as a significant cause of preterm birth. Emerging evidence indicates that Quercetin, a naturally occurring polyphenol, may offer protective benefits against preterm labor by inhibiting inflammatory pathways and improving fetal survival rates [30]. Several studies have also investigated the effects of Quercetin on pregnancy and miscarriage in vivo. Wu et al. conducted a study using an LPS-induced SA mouse model, revealing that gavaging with low-dose Quercetin (0.25 mg/mL) has the potential to decrease embryo loss rates and moderately increase the average embryo weight. The researchers highlighted that Quercetin may promote the expulsion or resorption of non-viable embryos post-miscarriage and support the uterus in returning to its pre-pregnancy condition [31]. In a murine model of LPS-induced preterm labor, the administration of Quercetin at a dose of 90 mg/kg via oral gavage significantly reduced the incidence of preterm labor by 63.5% and improved the neonatal survival rates to 83.76% [32]. Mechanistically, Quercetin achieved these protective effects by inhibiting the activation of Nuclear Factor Kappa B (NF-κB) and Activator protein 1 (AP-1), two critical transcription factors involved in mediating inflammatory responses. The study revealed that Quercetin downregulated the expression of NF-κB/P65, AP-1/C-JUN, Cyclooxygenase-2/Prostaglandin-Endoperoxide Synthase 2 (COX-2/PTGS2), and Interleukin 6 (IL-6) at both the mRNA and protein levels in the myometrium. This suppression of inflammatory pathways contributed to reduced uterine inflammation and played a key role in preventing the onset of preterm labor [33]. Liu et al. investigated the mechanisms underlying Quercetin’s protective effects using a pregnant mouse model exposed to PM2.5. Their research demonstrated that gavaged Quercetin (100 mg/kg) effectively prevents miscarriage by suppressing the expression of pro-inflammatory cytokines, such as IL-6 and Interleukin 8 (IL-8), while upregulating the levels of Heme Oxygenase-1 (HO-1) in peripheral blood [34]. Network pharmacology and UPLC-Q/TOF-MS analyses identified Quercetin as a key active component in BYLY, highlighting its potential role in enhancing pregnancy viability. In in vitro trophoblast models, Quercetin (2 μM) was shown to counteract hypoxia-induced apoptosis, mitochondrial dysfunction, and impaired cell proliferation, all of which are critical factors in recurrent spontaneous abortion [35] pathology [36]. Further mechanistic studies have revealed that Quercetin regulates the balance between mitochondrial fission and fusion by downregulating Drp1 expression through miR-34a-5p modulation, thereby supporting placental cell survival and function [37]. Notably, Quercetin’s potential benefits have been explored in clinical trials. A recent clinical trial involving 480 patients with recurrent SA demonstrated that a quadri-combination therapy (BYLY: Bushen, Yiqi, Lixue, Yangtai) significantly lowered early miscarriage rates when it used alongside duphaston, outperforming either treatment administered alone [36]. Despite the limited number of human clinical studies, these findings highlight Quercetin’s diverse roles in modulating immune responses, hormonal balance, and cellular processes. However, some studies have reported inconsistent results, indicating that Quercetin may also lead to side effects. For instance, doses of 50 μM of Quercetin were found to impair embryonic development in mice, causing a 20–30% reduction in morula and blastocyst formation rates [38]. Moreover, offspring of mice that were exposed to Quercetin (302 mg/kg feed) during pregnancy displayed a 40% increase in hepatic iron storage in adulthood, which was associated with alterations in cytokine expression levels, specifically IL-1β, IL-6, and IL-10 [38]. This prenatal exposure also induced hypermethylation of repetitive DNA elements, which may have implications for long-term gene expression modifications [39].

Thus, while the effects of Quercetin may vary depending on dosage and physiological context, these findings underscore the importance of conducting large-scale clinical trials in humans to further investigate its potential role in addressing recurrent spontaneous abortion and improving pregnancy outcomes.

## 4. Effect of Quercetin on Polycystic Ovary Syndrome

The ovarian microenvironment is essential for maintaining normal ovarian function and promoting female fertility [40]. Disruptions within this environment, as seen in PCOS, can have significant consequences. PCOS is a prevalent and complex endocrine disorder affecting approximately 5–20% of women of reproductive age, with the precise prevalence varying based on diagnostic criteria and the population studied [40,41]. It is a leading cause of anovulatory infertility, accounting for approximately 80% of cases [41] and is frequently associated with metabolic conditions such as insulin resistance (IR), obesity, and an increased risk of type 2 diabetes and cardiovascular disease [42,43,44]. PCOS is marked by a complex interplay of reproductive hormone imbalances. A hallmark of the condition is an elevated luteinizing hormone (LH)-to-follicle-stimulating hormone ratio [45]. In women with PCOS, the follicle-stimulating hormone [45] levels tend to be low to normal compared to those in normally cycling women during the early follicular phase of the menstrual cycle and lack the typical cyclic fluctuations necessary for ovulation. This hormonal dysregulation contributes to the development of enlarged, polycystic ovaries and clinical manifestations, including menstrual irregularities, infertility, and hirsutism [46]. Emerging evidence highlights the significant roles of inflammation and oxidative stress in PCOS pathophysiology. Oxidative stress and inflammation in PCOS operate within a self-perpetuating cycle. Specifically, oxidative stress can induce inflammation through the activation of NF-κB, a key transcription factor that promotes the expression of inflammatory cytokines such as Tumor Necrosis Factor-alpha (TNF-α) and IL-6 [47,48]. Inflammation, in turn, can amplify oxidative stress by enhancing ROS production [48]. This reciprocal interplay exacerbates IR and hyperandrogenemia, both of which are central to PCOS pathophysiology [48,49]. Furthermore, PCOS development is often linked to disruptions in key signaling pathways like Phosphoinositide 3 Kinase/Akt (PI3K/Akt), Transforming Growth Factor β/Suppressor of Mothers against Decapentaplegic (TGF-β/Smads), Wnt/β-catenin, and Hippo/YAP, which play a significant role in the disease’s progression [50]. Since, there is no standardized treatment for PCOS [41]. Clinical management focuses on personalized medication tailored to individual symptoms, particularly hormonal treatments. However, these therapies frequently have common side effects. For example, anti-estrogen medications, such as clomiphene citrate, are frequently used as the first-line treatment for anovulatory infertility in women with PCOS. It is important to note that obese women with PCOS may experience reduced effectiveness and lower pregnancy rates when using these medications to induce ovulation [51]. Therefore, it is evident that current treatment options primarily address specific aspects of PCOS, and they come with notable limitations. Given Quercetin’s anti-inflammatory and antioxidant effects, it has been widely studied as a possible PCOS treatment [6].

In vitro studies suggest that Quercetin protects granulosa cells from oxidative stress and apoptosis induced by various stressors. Quercetin treatment also reduced ROS levels and increased antioxidant and anti-apoptotic protein expression in ovary cells [52]. For instance, in hydrogen peroxide-induced oxidative stress in bovine granulosa cells, 10 uM of granulosa decreased the ROS levels, while it increased NF-E2-related factor 2 (Nrf2), thereby repairing cell damage [53]. Adding 2 ug/mL Quercetin to the maturation media enhanced the bovine oocytes’ quality and protected them from early apoptosis. The Quercetin-enriched media also strengthened cumulus cells, reducing their apoptosis and significantly increasing oocyte survival, with the upregulation of Octamer binding Transcription Factor 4 (OCT-4) and the receptors for the Insulin-like Growth Factor (IGF2R) and B-cell Lymphoma-2 (Bcl-2) proteins [54]. Several studies have also investigated Quercetin’s effects on PCOS in vivo. Wang et al. found that 2 mL of Quercetin (100 mg/kg) lowered the levels of inflammatory cytokines such as Interleukin-1 beta (IL-1β), IL-6, and TNFα, decreased NF-κB activation (including the nuclear translocation of NF-κBp65), and significantly reduced the phosphorylation of Insulin Receptor Substrate-1 (IRS-1) tyrosine in Dehydroepiandrosterone (DHEA)-induced PCOS in female Wistar rats. These results suggest Quercetin’s mechanism of action involves inhibiting the TLR/NF-κB signaling pathway, thereby improving the ovarian inflammatory microenvironment in PCOS [55]. Another study showed that the levels of the antioxidant enzymes Superoxide Dismutase (SOD), catalase, Glutathione-S-Transferase (GST), and reduced glutathione were significantly lower in PCOS rats. However, 100 mg/kg BW Quercetin significantly increased the levels of these enzymes in DHEA-induced PCOS in Wistar rats [56]. Quercetin treatment (30 mg/kg) in letrozole-induced PCOS in female Sprague Dawley (SD) rats led to a notable reduction in both the ovarian and cystic follicle diameters. Additionally, Quercetin demonstrated strong antioxidant properties, contributing to the recovery of ovarian cysts and promoting the health of follicles. Quercetin markedly regulated steroidogenesis, decreasing the testosterone and estradiol levels and increasing the progesterone levels [57]. In DHEA-induced PCOS rat models, oral administration with Quercetin (25 mg/kg) improved ovarian follicle development (increased preantral, antral, and preovulatory follicles and corpora lutea) and reduced follicular atresia and cyst formation, and modulated apoptosis markers (Bax and Bcl-2) in the ovaries. Additionally, Quercetin lowered the serum free testosterone and the LH/FSH ratio, mirroring the effects of metformin [58]. Shah et al. demonstrated that 125 mg/kg Quercetin in letrozole-induced PCOS Parkes strain mice models effectively normalized the elevated LH/FSH ratios and boosted estrogen levels. Notably, the study revealed that Quercetin played a vital role in preserving ovarian follicles by promoting the corpus luteum regeneration and eliminating cystic follicles, which ultimately helped reset the estrous cycle [59]. In a study using a DHEA-induced PCOS rat model, gavage daily administration of 2 mL of a quercetin solution (100 mg/kg) reduced serum testosterone, estradiol, LH, and the LH/FSH ratio, while it increased serum FSH. These effects were similar to those observed in a separate study using metformin in a similar DHEA-induced PCOS rat model [60]. Another study found that a Quercetin-rich extract from bitter melon could act as an insulin sensitizer and enhance ovulation, potentially aiding in the management of letrozole-induced PCOS in Swiss albino female rats. The hormonal analysis showed significant reductions in the LH, insulin, and testosterone levels in the treatment groups, along with improved FSH levels [61]. Hyperglycemia a common comorbidity of PCOS, primarily driven by insulin resistance. Research also indicates that bitter melon may possess hypoglycemic properties [62]. In a letrozole-induced PCOS rat model, *Fagonia indica* extract, rich in quercetin, improved PCOS symptoms. This plant extract reduced body weight, ovarian cysts, and improved follicular growth. Furthermore, it restored hormonal balance, lipid profiles, liver function markers, and increased antioxidant enzyme levels [63].

Most research on Quercetin’s effects utilizes animal models; however, there are few studies involving human patients with PCOS. This is significant considering that adiponectin levels are often reduced in individuals with PCOS. In this regard, Rezvan et al. Showed the effects of oral Quercetin supplementation in women with PCOS. Quercetin was effective on insulin sensitivity and hormone levels in women with PCOS. Compared to a placebo group, 1 g daily Quercetin supplementation slightly increased the total and high-molecular-weight adiponectin levels, significantly reduced the testosterone and LH levels, and also lowered insulin resistance (HOMA-IR) levels [64]. A randomized, double-blind, placebo-controlled trial investigated the effects of 1000 mg/day quercetin supplementation were investigated in overweight and obese women with PCOS. The study found that quercetin significantly reduced plasma resistin concentrations, mRNA levels, testosterone, and LH levels compared to the placebo group. Although fasting blood glucose, insulin, and HOMA-IR levels were decreased in the quercetin group, these changes were not statistically significant compared to the placebo [65]. Vaez et al. also found that Quercetin (500 mg) reduced inflammatory markers and LH levels in women with PCOS, while also improving the oocyte and embryo quality and increasing pregnancy rates [66]. In a study, researchers used network pharmacology and molecular docking to examine the mechanisms of BUSHEN HUATAN (BHHD), a Chinese medicine which consists of eight different herbs for treating PCOS patients. They found that it may improve insulin resistance and hormonal imbalances through various components and pathways, with Quercetin identified as a key component in its effectiveness [67]. Moreover, Yi-Jing Decoction (YJD), a traditional Chinese medicine, has been utilized for patients with PCOS. Research has identified quercetin as one of its potentially beneficial constituents. The findings suggest that YJD may treat PCOS by regulating levels of androgens and insulin, as well as by reducing inflammation. These effects are thought to result from interactions with key genes that are implicated in the pathogenesis of PCOS [68]. Notably, studies found no serious adverse events, reporting only mild gastrointestinal discomfort [69]. While these trials provided valuable insights into the therapeutic role of quercetin, they did not comprehensively assess broader clinical outcomes [6].

**Figure 1:**
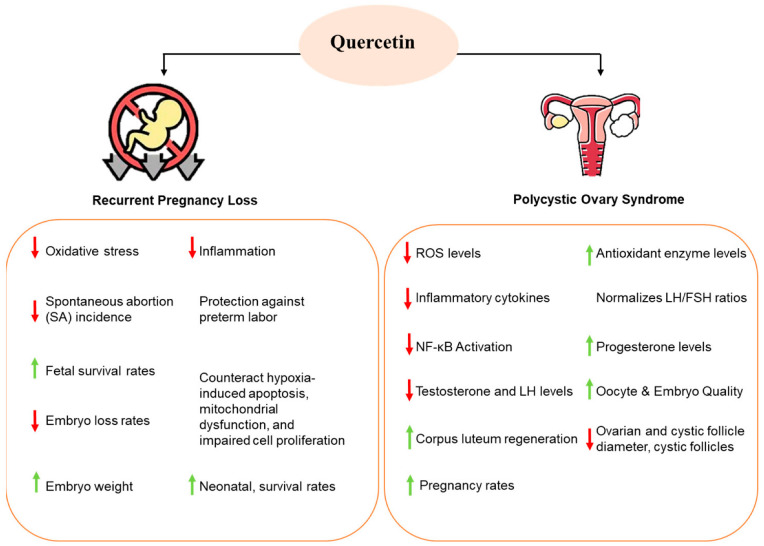
Effect of Quercetin on PCOS and pregnancy loss: potential mechanisms by which Quercetin alleviates PCOS symptoms and improves pregnancy outcomes.

Collectively, Quercetin shows promise as a PCOS treatment due to its antioxidant and anti-inflammatory properties, supported by positive in vitro and animal studies. However, larger clinical trials are needed to confirm its efficacy and optimal dosage (Figure 1).

## 5. Effect of Quercetin on Gynecological Cancers

Gynecological cancers including ovarian, endometrial, and cervical cancers pose a major global health challenge for women [70,71]. Despite advances in conventional treatments such as surgery, radiotherapy, and chemotherapy, these approaches often suffer from significant side effects and variable efficacy among patients [72,73]. This has driven growing interest in the potential of dietary interventions, particularly functional foods rich in plant bioactive compounds, to complement standard cancer therapies. Epidemiological studies have linked dietary patterns high in plant-based compounds to a reduced risk of various cancers [74,75]. As Quercetin has garnered significant interest for its potent anticancer properties, the following sections explore its effects on ovarian, cervical, and endometrial cancer. A summary of these effects is presented in Table 1.

### 5.1. Cervical Cancer

Cervical cancer is the fourth most frequently diagnosed malignancy in women and the fourth leading cause of cancer-related deaths worldwide [74]. In 2022, more than 662,000 new cases were reported globally, with approximately 349,000 deaths, highlighting its significant impact on female mortality [93]. Chronic inflammation and oxidative stress are recognized as key drivers in cervical carcinogenesis, contributing to processes such as cell proliferation, dysregulation of the cell cycle, migration, and apoptosis, and even epigenetic alterations [94,95,96]. Inflammation, while critical for tissue repair and immune defense, becomes detrimental when sustained over a prolonged period. In the context of cervical cancer, persistent inflammation, often initiated by human papillomavirus (HPV) infection, leads to the continuous secretion of pro-inflammatory cytokines such as TNF-α, Interferon gamma (IFN-γ), IL-1, and IL-8. In addition, molecules including Hypoxia-Inducible Factor (HIF), Inducible Nitric Oxide Synthase (iNOS), COX, Matrix Metalloproteinases (MMPs), and TGF-β, along with the activation of signaling pathways like NF-κB and Janus Kinase/signal Transducers and Activators of Transcription (JAK/STAT) [97], collectively promote tumor initiation, transformation, progression, and metastasis [98,99,100].

Quercetin has emerged as a promising natural agent in cervical cancer prevention and treatment due to its potent anti-inflammatory [101] and antioxidant [102] properties. Several in vitro studies using cervical cancer cell lines, such as SiHa Cells [76] and Hela cells [77], have demonstrated that Quercetin can suppress cell viability in a dose-dependent manner. It is observed that Quercetin treatment induces G2/M phase cell cycle arrest and triggers mitochondrial apoptosis through a p53-dependent mechanism [78,103]. This apoptotic effect is further reinforced by Quercetin’s capacity to downregulate key NF-κB family members (p50 and p65) and inhibit the NF-κB-mediated transcription of anti-apoptotic genes, thereby disrupting survival pathways and sensitizing cancer cells to programmed cell death [104].

Moreover, Quercetin has been shown to modulate the expression of inflammatory mediators involved in cervical carcinogenesis. In HeLa cells, Quercetin treatment (25–50 µM) leads to the decreased expression of Chemokine (C-X-C motif) Ligand 8 (CXCL8), MYC Proto-Oncogene, BHLH Transcription Factor (MYC), IL-2, and IL-1A, molecules that play crucial roles in sustaining the inflammatory microenvironment and promoting cancer progression [101]. These in vitro results indicate that Quercetin may disrupt inflammatory and survival pathways crucial for cervical cancer progression. Given its demonstrated positive effects in several in vitro studies, Quercetin shows promise as a functional food ingredient to support female reproductive health. Moreover, Quercetin has been reported to influence the epidermal growth factor receptor (EGFR) pathway. While several studies have indicated that Quercetin downregulates EGFR expression, thereby impeding cancer cell proliferation, some evidence suggests that Quercetin may also activate specific phosphorylation sites (such as Tyr1068) on EGFR, potentially diminishing its anticancer efficacy [76,105]. This contradictory evidence highlights the complexity of Quercetin’s molecular interactions and emphasizes the need for further research to clarify its precise role in EGFR signaling within cervical cancer cells.

In vivo studies, though limited in cervical cancer-specific models, support Quercetin’s anti-inflammatory and antioxidant effects observed in vitro. For example, Wei et al. investigated Quercetin’s effects on cervical cancer growth in a nude mouse model. The study found that Quercetin, at doses of 50 and 100 mg/kg administered orally, significantly inhibited tumor growth and altered the ultrastructure of tumor endothelial cells [79]. In addition, another study aiming to overcome Quercetin’s poor water solubility encapsulated Quercetin in PEGylated Liposomes (PEG-Que-NLs). The optimized formulation demonstrated a slow-release profile and significantly enhanced cytotoxicity against HeLa cells in vitro compared to those of free Quercetin and conventional liposomes. Furthermore, in vivo experiments in this study using U14 cervical carcinoma-bearing mice showed that intravenous injection of PEG-Que-NLs resulted in a significantly higher tumor inhibition rate than that achieved with free Quercetin or non-PEGylated liposomes, highlighting the potential of this delivery system for cervical cancer treatment [80].

Despite its potential benefits in cervical cancer, no clinical trials specifically examining Quercetin’s efficacy in this disease were identified in the search results. However, considering Quercetin’s safety profile and observed effects in other cancers, future clinical investigations are warranted. Well-designed clinical studies are essential for determining whether Quercetin, administered as a dietary supplement or through Quercetin-rich functional foods, can improve treatment outcomes for cervical cancer patients.

### 5.2. Ovarian Cancer

Ovarian cancer (OC) ranks as the third most prevalent and the deadliest cancer affecting the female reproductive system. Approximately 70% of patients are diagnosed at advanced stages, characterized by the presence of distant metastases [106]. Researchers have focused on diet quality as a promising option for therapy because of the fewer side effects associated with it [107].

Although the connections between diet, metabolism, and ovarian cancer are not fully clear, many bioactive compounds in functional foods possess antioxidant and anti-inflammatory properties. Therefore, functional foods may help prevent DNA damage and suppress the uncontrolled growth of cancer cells [108,109]. Regarding Quercetin’s antioxidant and anti-inflammatory properties, extensive experimental evidence has suggested that Quercetin can inhibit ovarian cancer cell proliferation and induce apoptosis, while also enhancing chemosensitivity and radiosensitivity and overcoming Cisplatin resistance [81,83,110,111,112,113,114].

For instance, Ren et al. examined Quercetin’s effects on the human ovarian cancer cell line SKOV-3. Their study found that treating these cells with Quercetin (0–30 mg/mL) led to a dose- and time-dependent reduction in cell proliferation. In addition, they discovered that Quercetin not only triggered apoptosis but also caused the cells to arrest in the G0/G1 phase of the cell cycle, with fewer cells progressing to the G2/M phase. Higher doses of Quercetin were associated with reduced levels of survivin, a protein that normally helps cells evade apoptosis [81]. In addition, a recent in vitro study by Teekaraman et al. explored the Quercetin’s anticancer on the PA-1 human metastatic ovarian cancer cell line. In their study, treating PA-1 cells with Quercetin at concentrations between 0 and 200 μM for 24 h led to a dose-dependent decrease in cell viability, with the most pronounced effects observed at 50 and 75 μM. On a molecular level, this treatment shifted the balance between apoptotic regulators by reducing anti-apoptotic proteins (Bcl-2 and Bcl-xL) while increasing pro-apoptotic markers (caspase-3, caspase-9, Bax, and cytochrome c). These findings suggest that Quercetin promotes cell death via the mitochondrial apoptotic pathway, underscoring its potential to inhibit metastatic ovarian cancer cell growth [82]. These pro-apoptotic and anti-proliferative effects have been observed in other lines as well, including OVCAR-3 [84], TOV-112D [85], and A2780 [83], indicating Quercetin’s ability to induce ovarian cancer cell death in vitro [115].

Beyond its direct cytotoxicity, Quercetin modulates key inflammatory and oxidative stress pathways in OC cells. Hasan et al. examined the impact of Quercetin on the ovarian adenocarcinoma SKOV-3 cell line and its cisplatin-resistant variant (SKOV-3/CDDP). They reported that treating the resistant cells with 100 μM Quercetin, which notably reduced cell viability, led to a marked suppression of genes encoding key antioxidant enzymes (SOD2, CAT, GPX1, and HO-1), the transcription factor Nrf2, and critical kinases in the Phosphoinositide 3 Kinase/Akt/mammalian target of Rapamycin (PI3K/Akt/mTOR) signaling pathway [86]. In their next study, Hasan et al. demonstrated that pre-treatment with Quercetin re-sensitizes cisplatin-resistant ovarian cancer SKOV-3/CDDP cells by suppressing the overexpression of antioxidant enzymes and the PI3K/Akt/mTOR signaling pathway, both of which are linked to cisplatin resistance. They showed that optimal Quercetin dosing (15–100 µM) not only arrests the cell cycle but also induces apoptosis through a pro-oxidant effect by inhibiting the Thioredoxin (Trx/TrxR) system, thereby increasing ROS production and activates the mitochondrial apoptotic cascade, as evidenced by the cleavage of caspases and Poly ADP-ribose polymerase (PARP) [87].

In vivo studies using animal models largely corroborate these in vitro findings. For instance, Li et al. demonstrated that, in ovarian cancer cell lines, low concentrations of Quercetin (5–30 μM) can significantly reduce the cytotoxic effects of chemotherapy drugs such as cisplatin, taxol, pirarubicin, and 5-fluorouracil, whereas higher doses (40–100 μM) are pro-apoptotic. This antagonistic effect is mediated by Quercetin’s capacity to mitigate ROS-induced injury and enhance endogenous antioxidant enzyme expression, ultimately diminishing drug-induced cell death. These in vitro results were corroborated in a xenograft mouse model, where a low-dose Quercetin pre-treatment (20 mg/kg, oral administration) reduced cisplatin’s efficacy by alleviating oxidative damage in tumor tissues, suggesting that Quercetin supplementation during ovarian cancer therapy may compromise treatment outcomes [114].

Clinical trial evidence for Quercetin’s efficacy in OC remains limited. An early Phase I trial evaluating intravenous Quercetin in advanced cancers, which included an ovarian cancer patient, reported acceptable safety profiles and an anecdotal reduction in the CA-125 tumor marker. Despite promising experimental findings, no subsequent Phase II trials have specifically evaluated the efficacy of quercetin in ovarian cancer, revealing a notable gap in the translation of research to clinical practice [88]. Numerous in vitro and in vivo studies have demonstrated that quercetin exerts multifaceted anticancer effects in ovarian cancer, including pro-apoptotic, anti-proliferative, anti-inflammatory, and anti-metastatic mechanisms. However, in contrast to earlier research suggesting the beneficial effects of quercetin, an experimental study found that quercetin may diminish the efficacy of certain antineoplastic agents, such as cisplatin, 5-fluorouracil, taxol, and pirarubicin. At low concentrations, quercetin displayed anti-apoptotic properties, reducing ROS-mediated damage from these drugs and increasing the activity of antioxidant enzymes in tumor cells [114]. To validate these findings in human populations, well-designed clinical trials are essential, as are strategies to optimize dosing and delivery systems to enhance quercetin’s low bioavailability.

### 5.3. Endometrial Cancer

Endometrial cancer (EC) represents the most prevalent form of gynecological cancer in developed countries, predominantly affecting postmenopausal women. Several risk factors have been identified, including obesity, nulliparity, late menopause, and specific genetic predispositions [116,117]. Treatment strategies for EC typically involve a combination of surgery, radiation, chemotherapy, and hormone therapy, determined by the cancer’s stage and subtype [118]. However, the increasing incidence of resistance to conventional therapies has intensified the focus on developing effective preventive measures and complementary treatments. This context has positioned functional foods as promising candidates for cancer prevention and management [119].

Quercetin has garnered attention for its potential chemopreventive and therapeutic effects in EC. In an in vitro study, Scambia et al. investigated Quercetin’s ability to inhibit colony formation in nine primary gynecological tumors (four ovarian and five endometrial). Quercetin (dissolved in solvent) alone exhibited a dose-dependent suppression of tumor cell proliferation (0.01–10 μM) [89]. In another study, Yang and Ma explored how Quercetin inhibited the EC cell development by targeting the Expression of Activating Transcription Factor 5 (ATF5)/JUN/PI3K/AKT/mTOR pathway and enhancing autophagy. Using network pharmacology and cellular experiments with the Ishikawa and HEC-1 A cell lines, they demonstrated that Quercetin suppresses cell proliferation, invasion, and migration while promoting apoptosis. These antitumor effects were closely linked to autophagy modulation through PI3K/AKT/mTOR pathway downregulation, highlighting Quercetin’s potential as a therapeutic agent for EC [90]. Zhang et al. conducted both in vitro and in vivo studies to examine how the flavonoids kaempferol and Quercetin, which directly bind to Nuclear Receptor 4A1 (NR4A1), can hinder the advancement of endometriosis. By inhibiting NR4A1, these compounds reduced the proliferation of human endometriotic epithelial and stromal cells, as well as of Ishikawa EC cells, through suppressing multiple growth and survival pathways (EGFR/c-Myc/survivin, mTOR) and fibrotic signaling, while also modifying oxidative stress pathways. In in vivo experiments using a mouse model of endometriosis, kaempferol and Quercetin (100 mg/kg/day, oral administration) effectively decreased the endometriotic lesion size without affecting the body weight, highlighting their potential as a nutritional therapy for endometriosis [91]. Li et al. conducted an in vitro study on HEC-1-A EC cells to evaluate Quercetin’s impact on cell viability, proliferation, and apoptosis, and the possible role of ferroptosis in these processes. Their results showed that 100 µM Quercetin inhibited proliferation and migration, induced cell cycle arrest, and promoted apoptosis of HEC-1-A cells. Furthermore, Quercetin’s antitumor effects appeared to be strongly linked to triggering ferroptosis, suggesting a potential therapeutic avenue for EC [92]. Nevertheless, the use of Quercetin in EC raises concerns due to its estrogenic properties. It has shown that Quercetin can produce significant estrogenic effects, increasing serum estradiol levels by up to 22.48%. Since EC is typically estrogen-sensitive, this estrogenic activity may diminish its potential anticancer benefits for certain patients [120]. Given EC’s multifaceted nature, a personalized approach that considers genetic and hormonal factors could further refine Quercetin’s clinical application. However, the absence of clinical trials investigating quercetin’s effects on endometrial cancer is notable.

## 6. Limitations and Challenges of Quercetin Usage

Promising findings from clinical trial studies have been observed, suggesting that quercetin may have beneficial effects on various conditions such as hyperuricemia [121], PCOS [64], obesity [122], and blood pressure [123]. However, there is a notable lack of clinical research focusing specifically on gynecological cancers and pregnancy-related issues, which is significant as it limits our understanding of quercetin’s efficacy in these critical domains. Preclinical studies have identified several limitations that impede the therapeutic application of quercetin in the female reproductive system. One of the most important challenges is its low bioavailability, which restricts optimal therapeutic outcomes [124]. Joseph et al. demonstrated that quercetin’s clinical application is hindered by poor water solubility and limited oral absorption, prompting the development of advanced hybrid hydrogel delivery systems designed to enhance its bioavailability [125]. Furthermore, studies have shown that quercetin undergoes rapid phase II metabolism, primarily through glucuronidation and sulfation shortly after absorption, significantly affecting its systemic availability and therapeutic window [126,127,128]. In response to these challenges, researchers are exploring innovative drug delivery systems, including quercetin-loaded gels, polymeric micelles [129], nanoparticles [129], and glucan-quercetin conjugates [130]. However, a notable methodological weakness has been identified in these studies due to the lack of standardization concerning the source of quercetin utilized [131]. Additionally, mostly studies which are reviewed in this article, have been conducted over short durations, ranging from weeks to a few months, which limits the evaluation of long-term efficacy and safety regarding reproductive disorders. This limitation is particularly concerning given the substantial discrepancy between pharmacological doses used in research, up to 945 mg/m² intravenously [88]. In contrast, a study conducted in Japan found that the median intake in a typical Western diet is closer to 10 mg/day, with primary sources being onions, apples, and green tea [132]. This disparity underscores the critical distinction between quercetin’s role as a dietary component and its potential as a pharmaceutical agent. Addressing these gaps highlights an urgent need for further large-scale, randomized controlled trials to establish quercetin’s clinical efficacy and safety while clarifying optimal dosages and administration routes in women’s reproductive health.

## 7. Conclusions

Quercetin demonstrates compelling potential in managing various female reproductive health conditions, including pregnancy-related challenges, PCOS, and gynecological cancers. Its antioxidant, anti-inflammatory, and hormone-modulating properties have been highlighted across numerous in vitro and in vivo studies, with limited clinical trials indicating early but promising benefits. Moreover, incorporating quercetin into diets could offer a practical, patient-friendly strategy to enhance the substance’s bioavailability and therapeutic efficacy. Future investigations should focus on well-structured clinical trials to confirm these findings, optimize dosing, and address potential interactions with conventional therapies, ultimately advancing Quercetin’s role as a valuable component in women’s reproductive healthcare.

## Figures and Tables

**Table 1:** Quercetin’s effect on gynecological cancers.

Cancer Type	Sample/Model	Effect of Quercetin	Study Type	References
Cervical	HeLa, SiHa cells	↓ cell viability (dose-dependent), G2/M arrest, p53-dependent mitochondrial apoptosis, ↓ NF-κB (p50, p65), ↓ CXCL8, MYC, IL-2, IL-1A	In vitro	[76,77,78]
Cervical	Nude mouse model (xenograft)	↓ tumor growth, alters tumor endothelial ultrastructure, potential anti-angiogenic effects	In vivo	[79]
Cervical	U14 tumor-bearing mice + PEG-Que-NLs	Improved cytotoxicity vs. free quercetin; higher tumor inhibition rate with PEGylated liposomes	In vivo	[80]
Ovarian	SKOV-3 cells	Dose/time-dependent ↓ in cell proliferation, G0/G1 arrest, ↓ survivin, ↑ apoptosis	In vitro	[81]
Ovarian	PA-1 cell line (human metastatic)	↓ Bcl-2, Bcl-xL; ↑ Bax, caspase-3/9, cytochrome c → promotes mitochondrial apoptosis	In vitro	[82]
Ovarian	OVCAR-3 TOV-112D A2780	Overcomes cisplatin resistance and radiosensitization; pro-apoptotic, anti-proliferative, anti-inflammatory	In vitro	[83,84,85]
Ovarian	SKOV-3 /mouse xenograft	Low-dose Quercetin reduces chemo cytotoxicity; high dose is pro-apoptotic (↑ antioxidant enzymes, ↓ oxidative damage)	In vitro In vivo	[83]
Ovarian	Cisplatin-resistant SKOV-3/CDDP	Re-sensitizes resistant cells to cisplatin, blocks PI3K/Akt/mTOR, ↓ Nrf2 and SOD2, promotes mitochondrial apoptosis, pro-oxidant effect	In vitro	[86,87]
Ovarian	Phase I trial (advanced cancers)	IV quercetin safe, anecdotal ↓ CA-125 in an ovarian cancer patient; no follow-up Phase II specific to OC	Clinical (Phase I)	[88]
Endometrial	Tumors	Quercetin (0.01–10 μM) → dose-dependent suppression of colony formation	In vitro	[89]
Endometrial	Ishikawa, HEC-1 A cells	↓ proliferation, invasion, migration;	In vitro	[90]
		↑ apoptosis; modulates ATF5/JUN/PI3K/AKT/mTOR, induces autophagy		
Endometrial	Human endometriotic epithelial and stromal cells) + Animal model (mouse)	↓ proliferation, suppressed multiple pathways (EGFR/c-Myc/survivin, mTOR), ↓ lesion size in mouse model	In vitro In vivo	[91]
Endometrial	HEC-1-A cells	↓ proliferation, migration; ↑ apoptosis, cell cycle arrest; triggers ferroptosis	In vitro	[92]

NF-Κb—Nuclear Factor Kappa B; CXCL8—C-X-C motif chemokine ligand 8; IL-2—interleukin 1; IL-1A—interleukin 1A; PI3K/Akt/mTOR—phosphoinositide 3 kinase; Nrf2—nuclear factor erythroid 2–related factor 2; SOD2—superoxide dismutase 2; CA-125—cancer antigen 125; OC—ovarian cancer; ATF5/JUN/PI3K/AKT/mTOR—activating Transcription Factor 5; EGFR—epidermal growth factor receptor; ↓—decrease; ↑— increase; →leads to or results in.

## Data Availability

No new data were created or analyzed in this study. Data sharing does not apply to this article.

## References

[1] Szilágyi A., Szabó I. (2003). Endocrine Characteristics of Polycystic Ovary Syndrome (PCOS).

[2] Keyvani V., Kheradmand N., Navaei Z.N., Mollazadeh S., Esmaeili S.-A. (2023). Epidemiological trends and risk factors of gynecological cancers: An update. Med. Oncol..

[3] Laijawala R.A. (2024). Recurrent Pregnancy Loss: Immunological aetiologies and associations with mental health. Brain Behav. Immun. -Health.

[4] Madigan M., Karhu E. (2018). The role of plant-based nutrition in cancer prevention. J. Unexplored Med. Data.

[5] DeClercq V., Nearing J.T., Sweeney E. (2022). Plant-Based Diets and Cancer Risk: What is the Evidence?. Curr. Nutr. Rep..

[6] Pourteymour Fard Tabrizi F., Hajizadeh-Sharafabad F., Vaezi M., Jafari-Vayghan H., Alizadeh M., Maleki V. (2020). Quercetin and polycystic ovary syndrome, current evidence and future directions: A systematic review. J. Ovarian Res..

[7] Li Y., Yao J., Han C., Yang J., Chaudhry M.T., Wang S., Liu H., Yin Y. (2016). Quercetin, inflammation and immunity. Nutrients.

[8] Vašková J., Klepcová Z., Špaková I., Urdzík P., Štofilová J., Bertková I., Kľoc M., Rabajdová M. (2023). The importance of natural antioxidants in female reproduction. Antioxidants.

[9] Valentová K., Šíma P., Rybková Z., Křížan J., Malachová K., Křen V. (2016). (Anti)mutagenic and immunomodulatory properties of quercetin glycosides. J. Sci. Food Agric..

[10] Deepika, Maurya P.K. (2022). Health Benefits of Quercetin in Age-Related Diseases. Molecules.

[11] Ding J., Mei S., Cai M., Zhang D., Yu J. (2022). Integrated network pharmacology and clinical study to reveal the effects and mechanisms of bushen huoxue huatan decoction on polycystic ovary syndrome. Evid. -Based Complement. Altern. Med..

[12] Sharma H., Sen S., Singh N. (2005). Molecular pathways in the chemosensitization of cisplatin by quercetin in human head and neck cancer. Cancer Biol. Ther..

[13] Li J., Long H., Cong Y., Gao H., Lyu Q., Yu S., Kuang Y. (2021). Quercetin prevents primordial follicle loss via suppression of PI3K/Akt/Foxo3a pathway activation in cyclophosphamide-treated mice. Reprod. Biol. Endocrinol..

[14] Lai W.-F., Wong W.-T. (2022). Design and optimization of quercetin-based functional foods. Crit. Rev. Food Sci. Nutr..

[15] Kennedy D.A., Lupattelli A., Koren G., Nordeng H. (2013). Herbal medicine use in pregnancy: Results of a multinational study. BMC Complement. Altern. Med..

[16] Ożarowski M., Mikołajczak P.Ł., Kujawski R., Wielgus K., Klejewski A., Wolski H., Seremak-Mrozikiewicz A. (2018). Pharmacological effect of quercetin in hypertension and its potential application in pregnancy-induced hypertension: Review of in vitro, in vivo, and clinical studies. Evid. -Based Complement. Altern. Med..

[17] Tomkiewicz J., Darmochwał-Kolarz D. (2023). The diagnostics and treatment of recurrent pregnancy loss. J. Clin. Med..

[18] Melo P., Dhillon-Smith R., Islam M.A., Devall A., Coomarasamy A. (2023). Genetic causes of sporadic and recurrent miscarriage. Fertil. Steril..

[19] Carbonnel M., Pirtea P., de Ziegler D., Ayoubi J.M. (2021). Uterine factors in recurrent pregnancy losses. Fertil. Steril..

[20] Practice Committee of the American Society for Reproductive Medicine (2012). Evaluation and treatment of recurrent pregnancy loss: A committee opinion. Fertil. Steril..

[21] Hempstock J., Jauniaux E., Greenwold N., Burton G.J. (2003). The contribution of placental oxidative stress to early pregnancy failure. Hum. Pathol..

[22] Obeagu E., Obeagu G. (2024). Enhancing maternal and fetal well-being: The role of antioxidants in pregnancy. Elite J. Med. Sci..

[23] Araújo J.R., Correia-Branco A., Pereira A.C., Pinho M.J., Keating E., Martel F. (2013). Oxidative stress decreases uptake of neutral amino acids in a human placental cell line (BeWo cells). Reprod. Toxicol..

[24] Bartho L.A., Holland O.J., Moritz K.M., Perkins A.V., Cuffe J.S. (2019). Maternal corticosterone in the mouse alters oxidative stress markers, antioxidant function and mitochondrial content in placentas of female fetuses. J. Physiol..

[25] Umekawa T., Sugiyama T., Kihira T., Murabayashi N., Zhang L., Nagao K., Kamimoto Y., Ma N., Yodoi J., Sagawa N. (2008). Overexpression of thioredoxin-1 reduces oxidative stress in the placenta of transgenic mice and promotes fetal growth via glucose metabolism. Endocrinology.

[26] Webster J., Miller M., Vemulapalli R. (2008). Encephalitozoon cuniculi-associated placentitis and perinatal death in an alpaca (Lama pacos). Vet. Pathol..

[27] Safronova V., Matveeva N., Avkhacheva N., Sidel’nikova V., Van’ko L., Sukhikh G. (2003). Changes in regulation of oxidase activity of peripheral blood granulocytes in women with habitual abortions. Bull. Exp. Biol. Med..

[28] Yoshida K., Kusama K., Shinohara G., Sato S., Yoshie M., Tamura K. (2024). Quercetin stimulates trophoblast fusion via the mitochondrial function. Sci. Rep..

[29] Takashima M., Tanaka W., Matsuyama H., Tajiri H., Sakakibara H. (2021). Maternal quercetin consumption during pregnancy may help regulate total cholesterol/HDL-cholesterol ratio without effect on cholesterol levels in male progeny consuming high-fat diet. Nutrients.

[30] Wang D., Li X., Li Y., Wang R., Wang C., Li Y. (2024). New molecular mechanisms of quercetin in improving recurrent spontaneous abortion based on in-depth network pharmacology and molecular docking. Front. Chem..

[31] Wu S., Tian Y., Zhang Q., Fu Z., Lan H., Zhou X., Ma L., Lou Y. (2024). Protective effect of quercetin on lipopolysaccharide-induced miscarriage based on animal experiments and network pharmacology. Mol. Med. Rep..

[32] Lin X., Peng Q., Zhang J., Li X., Huang J., Duan S., Zhang W. (2020). Quercetin prevents lipopolysaccharide-induced experimental preterm labor in mice and increases offspring survival rate. Reprod. Sci..

[33] Dong J., Young P.J., Se T., Yahweh, Hwi L.M. Quercetin inhibits NF-κB and AP-1 activation through induction of heme oxygenase-1 and attenuation of Src mediated PI3K/Akt, p38 and c-jun N-terminal protein kinase phosphorylations in raw 264.7 cells. Proceedings of the 2012 Winter Symposium of the Korean Society for Laboratory Animal Science.

[34] Liu W., Zhang M., Feng J., Fan A., Zhou Y., Xu Y. (2017). The influence of quercetin on maternal immunity, oxidative stress, and inflammation in mice with exposure of fine particulate matter during gestation. Int. J. Environ. Res. Public Health.

[35] Ahmadi R., Ziaei S., Parsay S. (2016). Association between nutritional status with spontaneous abortion. Int. J. Fertil. Steril..

[36] Zhou J., Li L., Pan X., Wang J., Qi Q., Sun H., Li C., Wang L. (2022). The effect of a traditional Chinese quadri-combination therapy and its component quercetin on recurrent spontaneous abortion: A clinical trial, network pharmacology and experiments-based study. Front. Pharmacol..

[37] Wang H., Lin H., Kang W., Huang L., Gong S., Zhang T., Huang X., He F., Ye Y., Tang Y. (2023). miR-34a/DRP-1-mediated mitophagy participated in cisplatin-induced ototoxicity via increasing oxidative stress. BMC Pharmacol. Toxicol..

[38] Yu S., Long H., Lyu Q.F., Zhang Q.H., Yan Z.G., Liang H.X., Chai W.R., Yan Z., Kuang Y.P., Qi C. (2014). Protective effect of quercetin on the development of preimplantation mouse embryos against hydrogen peroxide-induced oxidative injury. PLoS ONE.

[39] Vanhees K., Godschalk R.W., Sanders A., van Waalwijk van Doorn-Khosrovani S.B., van Schooten F.J. (2011). Maternal quercetin intake during pregnancy results in an adapted iron homeostasis at adulthood. Toxicology.

[40] Greff D., Juhász A.E., Váncsa S., Váradi A., Sipos Z., Szinte J., Park S., Hegyi P., Nyirády P., Ács N. (2023). Inositol is an effective and safe treatment in polycystic ovary syndrome: A systematic review and meta-analysis of randomized controlled trials. Reprod. Biol. Endocrinol..

[41] Teede H.J., Misso M.L., Costello M.F., Dokras A., Laven J., Moran L., Piltonen T., Norman R.J. (2018). Recommendations from the international evidence-based guideline for the assessment and management of polycystic ovary syndrome. Hum. Reprod..

[42] Zhu T., Cui J., Goodarzi M.O. (2021). Polycystic ovary syndrome and risk of type 2 diabetes, coronary heart disease, and stroke. Diabetes.

[43] Shrivastava S., Conigliaro R.L. (2023). Polycystic ovarian syndrome. Med. Clin. N. Am..

[44] Zehravi M., Maqbool M., Ara I. (2022). Polycystic ovary syndrome and infertility: An update. Int. J. Adolesc. Med. Health.

[45] Tousizadeh S., Mohammadi-Moghadam F., Mohammadian-Hafshejani A., Sadeghi R. (2024). Comparison of zinc levels in mothers with and without abortion: A systematic review and meta-analysiss. Heliyon.

[46] Dapas M., Dunaif A. (2022). Deconstructing a syndrome: Genomic insights into PCOS causal mechanisms and classification. Endocr. Rev..

[47] Li W., Liu C., Yang Q., Zhou Y., Liu M., Shan H. (2022). Oxidative stress and antioxidant imbalance in ovulation disorder in patients with polycystic ovary syndrome. Front. Nutr..

[48] Mancini A., Bruno C., Vergani E., d’Abate C., Giacchi E., Silvestrini A. (2021). Oxidative stress and low-grade inflammation in polycystic ovary syndrome: Controversies and new insights. Int. J. Mol. Sci..

[49] Mazloomi S., Sheikh N., Sanoee Farimani M., Pilehvari S. (2021). Association of Prx4, total oxidant status, and inflammatory factors with insulin resistance in polycystic ovary syndrome. Int. J. Endocrinol..

[50] Wang K., Li Y. (2023). Signaling pathways and targeted therapeutic strategies for polycystic ovary syndrome. Front. Endocrinol..

[51] Legro R.S., Arslanian S.A., Ehrmann D.A., Hoeger K.M., Murad M.H., Pasquali R., Welt C.K. (2013). Diagnosis and treatment of polycystic ovary syndrome: An Endocrine Society clinical practice guideline. J. Clin. Endocrinol. Metab..

[52] Ma C., Xiang Q., Song G., Wang X. (2022). Quercetin and polycystic ovary syndrome. Front. Pharmacol..

[53] Khadrawy O.Z.S. (2019). Modulation of Nrf2-Mediated Oxidative Stress Response in Bovine Granulosa Cells and Preimplantation Embryos.

[54] Davoodian N., Kadivar A., Davoodian N., Ahmadi E., Nazari H., Mehrban H. (2022). The effect of quercetin in the maturation media on cumulus-granulosa cells and the developmental competence of bovine oocytes. Theriogenology.

[55] Wang Z., Zhai D., Zhang D., Bai L., Yao R., Yu J., Cheng W., Yu C. (2017). Quercetin Decreases Insulin Resistance in a Polycystic Ovary Syndrome Rat Model by Improving Inflammatory Microenvironment. Reprod. Sci..

[56] Olaniyan O.T., Bamidele O., Adetunji C.O., Priscilla B., Femi A., Ayobami D., Okotie G., Oluwaseun I., Olugbenga E., Mali P.C. (2020). Quercetin modulates granulosa cell mRNA androgen receptor gene expression in dehydroepiandrosterone-induced polycystic ovary in Wistar rats via metabolic and hormonal pathways. J. Basic Clin. Physiol. Pharmacol..

[57] Jahan S., Abid A., Khalid S., Afsar T., Shaheen G., Almajwal A., Razak S. (2018). Therapeutic potentials of Quercetin in management of polycystic ovarian syndrome using Letrozole induced rat model: A histological and a biochemical study. J. Ovarian Res..

[58] Mahmoud A.A., Elfiky A.M., Abo-Zeid F.S. (2022). The anti-androgenic effect of quercetin on hyperandrogenism and ovarian dysfunction induced in a dehydroepiandrosterone rat model of polycystic ovary syndrome. Steroids.

[59] Shah M.Z.u.h., Shrivastva V.k., Mir M.A., Sheikh W.M., Ganie M.A., Rather G.A., Shafi M., Bashir S.M., Ansari M.A., Al-Jafary M.A. (2023). Effect of quercetin on steroidogenesis and folliculogenesis in ovary of mice with experimentally-induced polycystic ovarian syndrome. Front. Endocrinol..

[60] Zheng S., Chen Y., Ma M., Li M. (2022). Mechanism of quercetin on the improvement of ovulation disorder and regulation of ovarian CNP/NPR2 in PCOS model rats. J. Formos. Med. Assoc..

[61] Hussain L., Aamir N., Hussain M., Asif M., Chauhdary Z., Manzoor F., Siddique R., Riaz M. (2022). Therapeutic investigation of standardized aqueous methanolic extract of bitter melon (*Momordica charantia* L.) for its potential against polycystic ovarian syndrome in experimental animals’ model: In vitro and in vivo studies. Evid. -Based Complement. Altern. Med..

[62] Joseph B., Jini D. (2013). Antidiabetic effects of Momordica charantia (bitter melon) and its medicinal potency. Asian Pac. J. Trop. Dis..

[63] Younas A., Hussain L., Shabbir A., Asif M., Hussain M., Manzoor F. (2022). Effects of fagonia indica on letrozole-induced polycystic ovarian syndrome (PCOS) in young adult female rats. Evid. -Based Complement. Altern. Med..

[64] Rezvan N., Moini A., Janani L., Mohammad K., Saedisomeolia A., Nourbakhsh M., Gorgani-Firuzjaee S., Mazaherioun M., Hosseinzadeh-Attar M.J. (2017). Effects of Quercetin on Adiponectin-Mediated Insulin Sensitivity in Polycystic Ovary Syndrome: A Randomized Placebo-Controlled Double-Blind Clinical Trial. Horm. Metab. Res..

[65] Khorshidi M., Moini A., Alipoor E., Rezvan N., Gorgani-Firuzjaee S., Yaseri M., Hosseinzadeh-Attar M.J. (2018). The effects of quercetin supplementation on metabolic and hormonal parameters as well as plasma concentration and gene expression of resistin in overweight or obese women with polycystic ovary syndrome. Phytother. Res..

[66] Vaez S., Parivr K., Amidi F., Rudbari N.H., Moini A., Amini N. (2023). Quercetin and polycystic ovary syndrome; inflammation, hormonal parameters and pregnancy outcome: A randomized clinical trial. Am. J. Reprod. Immunol..

[67] Hong Y.-L., Wu F. (2014). Effect of Bushen Huatan Recipe on the Akt signal pathway in polycystic ovarian syndrome model rats with insulin resistance: An experimental research. Zhongguo Zhong Xi Yi Jie He Za Zhi = Chin. J. Integr. Tradit. West. Med..

[68] Lin Y., Xiang L., Li X., Tang Q., Meng F., Chen W. (2023). Exploring the mechanism of Yi-Jing decoction in treating polycystic ovary syndrome by using network pharmacology. Curr. Med. Chem..

[69] Egert S., Bosy-Westphal A., Seiberl J., Kürbitz C., Settler U., Plachta-Danielzik S., Wagner A.E., Frank J., Schrezenmeir J., Rimbach G. (2009). Quercetin reduces systolic blood pressure and plasma oxidised low-density lipoprotein concentrations in overweight subjects with a high-cardiovascular disease risk phenotype: A double-blinded, placebo-controlled cross-over study. Br. J. Nutr..

[70] Zhu G., Li Z., Tang L., Shen M., Zhou Z., Wei Y., Zhao Y., Bai S., Song L. (2022). Associations of Dietary Intakes with Gynecological Cancers: Findings from a Cross-Sectional Study. Nutrients.

[71] Ferlay J., Colombet M., Soerjomataram I., Parkin D.M., Piñeros M., Znaor A., Bray F. (2021). Cancer statistics for the year 2020: An overview. Int. J. Cancer.

[72] Tiwary S., Hussain M. (2021). Functional foods for prevention and treatment of cancer. Asian. J. Pharm. Clin. Res..

[73] Serna-Thomé G., Castro-Eguiluz D., Fuchs-Tarlovsky V., Sánchez-López M., Delgado-Olivares L., Coronel-Martínez J., Molina-Trinidad E.M., de la Torre M., Cetina-Pérez L. (2018). Use of functional foods and oral supplements as adjuvants in cancer treatment. Rev. Investig. Clin..

[74] Saslow D., Solomon D., Lawson H.W., Killackey M., Kulasingam S.L., Cain J., Garcia F.A., Moriarty A.T., Waxman A.G., Wilbur D.C. (2012). American Cancer Society, American Society for Colposcopy and Cervical Pathology, and American Society for Clinical Pathology screening guidelines for the prevention and early detection of cervical cancer. CA Cancer J. Clin..

[75] Sak K. (2012). Chemotherapy and dietary phytochemical agesnts. Chemother. Res. Pract..

[76] Chen X., Xu P., Zhang H., Su X., Guo L., Zhou X., Wang J., Huang P., Zhang Q., Sun R. (2021). EGFR and ERK activation resists flavonoid quercetin-induced anticancer activities in human cervical cancer cells in vitro. Oncol. Lett..

[77] He C., Lu X., Li J., Shen K., Bai Y., Li Y., Luan H., Tuo S. (2021). The effect of quercetin on cervical cancer cells as determined by inducing tumor endoplasmic reticulum stress and apoptosis and its mechanism of action. Am. J. Transl. Res..

[78] Vidya Priyadarsini R., Senthil Murugan R., Maitreyi S., Ramalingam K., Karunagaran D., Nagini S. (2010). The flavonoid quercetin induces cell cycle arrest and mitochondria-mediated apoptosis in human cervical cancer (HeLa) cells through p53 induction and NF-κB inhibition. Eur. J. Pharmacol..

[79] Wei W., Liu T., Ding B., Cao S. (2015). Study on relationship of quercetin on cervical carcinoma in nude mice model of tumor growth and the expression of heparanase. Chin. J. Biochem. Pharm..

[80] Li J., Li Z., Gao Y., Liu S., Li K., Wang S., Gao L., Shi M., Liu Z., Han Z. (2021). Effect of a Drug Delivery System Made of Quercetin Formulated into PEGylation Liposomes on Cervical Carcinoma In Vitro and In Vivo. J. Nanomater..

[81] Ren M.X., Deng X.H., Ai F., Yuan G.Y., Song H.Y. (2015). Effect of quercetin on the proliferation of the human ovarian cancer cell line SKOV-3 in vitro. Exp. Ther. Med..

[82] Teekaraman D., Elayapillai S.P., Viswanathan M.P., Jagadeesan A. (2019). Quercetin inhibits human metastatic ovarian cancer cell growth and modulates components of the intrinsic apoptotic pathway in PA-1 cell line. Chem. -Biol. Interact..

[83] Zhou J., Gong J., Ding C., Chen G. (2015). Quercetin induces the apoptosis of human ovarian carcinoma cells by upregulating the expression of microRNA-145. Mol. Med. Rep..

[84] Luo H., Jiang B.-H., King S.M., Chen Y.C. (2008). Inhibition of cell growth and VEGF expression in ovarian cancer cells by flavonoids. Nutr. Cancer.

[85] Xintaropoulou C., Ward C., Wise A., Marston H., Turnbull A., Langdon S.P. (2015). A comparative analysis of inhibitors of the glycolysis pathway in breast and ovarian cancer cell line models. Oncotarget.

[86] Hasan A.A.S., Kalinina E.V., Tatarskiy V.V., Volodina Y.L., Petrova A.S., Novichkova M.D., Zhdanov D.D., Shtil A.A. (2022). Suppression of the Antioxidant System and PI3K/Akt/mTOR Signaling Pathway in Cisplatin-Resistant Cancer Cells by Quercetin. Bull. Exp. Biol. Med..

[87] Hasan A.A., Kalinina E., Nuzhina J., Volodina Y., Shtil A., Tatarskiy V. (2023). Potentiation of Cisplatin Cytotoxicity in Resistant Ovarian Cancer SKOV3/Cisplatin Cells by Quercetin Pre-Treatment. Int. J. Mol. Sci..

[88] Ferry D.R., Smith A., Malkhandi J., Fyfe D.W., deTakats P.G., Anderson D., Baker J., Kerr D.J. (1996). Phase I clinical trial of the flavonoid quercetin: Pharmacokinetics and evidence for in vivo tyrosine kinase inhibition. Clin. Cancer Res..

[89] Scambia G., Ranelletti F.O., Panici P.B., Piantelli M., Bonanno G., De Vincenzo R., Ferrandina G., Maggiano N., Capelli A., Mancuso S. (1992). Inhibitory effect of quercetin on primary ovarian and endometrial cancers and synergistic activity with cis-diamminedichloroplatinum(II). Gynecol. Oncol..

[90] Yang L., Ma H. (2025). Quercetin inhibits the biological activity of endometrial cancer by regulating autophagy: A network pharmacology analysis and cellular experimental validation. J. Funct. Foods.

[91] Zhang L., Mohankumar K., Martin G., Mariyam F., Park Y., Han S.J., Safe S. (2023). Flavonoids Quercetin and Kaempferol Are NR4A1 Antagonists and Suppress Endometriosis in Female Mice. Endocrinology.

[92] Li X., Zhu Q., Ma M., Guo H. (2022). Quercetin inhibits the progression of endometrial HEC-1-A cells by regulating ferroptosis—A preliminary study. Eur. J. Med. Res..

[93] Bray F., Laversanne M., Sung H., Ferlay J., Siegel R.L., Soerjomataram I., Jemal A. (2024). Global cancer statistics 2022: GLOBOCAN estimates of incidence and mortality worldwide for 36 cancers in 185 countries. CA Cancer J. Clin..

[94] Preci D.P., Almeida A., Weiler A.L., Franciosi M.L.M., Cardoso A.M. (2021). Oxidative damage and antioxidants in cervical cancer. Int. J. Gynecol. Cancer.

[95] Hemmat N., Bannazadeh Baghi H. (2019). Association of human papillomavirus infection and inflammation in cervical cancer. Pathog. Dis..

[96] Holub K., Biete A. (2019). Impact of systemic inflammation biomarkers on the survival outcomes of cervical cancer patients. Clin. Transl. Oncol..

[97] Gutiérrez-Hoya A., Soto-Cruz I. (2020). Role of the JAK/STAT pathway in cervical cancer: Its relationship with HPV E6/E7 oncoproteins. Cells.

[98] Suarez-Carmona M., Lesage J., Cataldo D., Gilles C. (2017). EMT and Inflammation: Inseparable Actors of Cancer Progression. Mol. Oncol..

[99] Yeung Y.T., Aziz F., Guerrero-Castilla A., Arguelles S. (2018). Signaling pathways in inflammation and anti-inflammatory therapies. Curr. Pharm. Des..

[100] Georgescu S.R., Mitran C.I., Mitran M.I., Caruntu C., Sarbu M.I., Matei C., Nicolae I., Tocut S.M., Popa M.I., Tampa M. (2018). New insights in the pathogenesis of HPV infection and the associated carcinogenic processes: The role of chronic inflammation and oxidative stress. J. Immunol. Res..

[101] Kedhari Sundaram M., Raina R., Afroze N., Bajbouj K., Hamad M., Haque S., Hussain A. (2019). Quercetin modulates signaling pathways and induces apoptosis in cervical cancer cells. Biosci. Rep..

[102] Alrawaiq N.S., Abdullah A. (2014). A review of flavonoid quercetin: Metabolism, bioactivity and antioxidant properties. Int. J. Pharm.Tech. Res..

[103] Clemente-Soto A.F., Salas-Vidal E., Milan-Pacheco C., Sánchez-Carranza J.N., Peralta-Zaragoza O., González-Maya L. (2019). Quercetin induces G2 phase arrest and apoptosis with the activation of p53 in an E6 expression-independent manner in HPV-positive human cervical cancer-derived cells. Mol. Med. Rep..

[104] Deng S., Yuan P., Sun J. (2024). The role of NF-κB in carcinogenesis of cervical cancer: Opportunities and challenges. Mol. Biol. Rep..

[105] Pani S., Mohapatra S., Sahoo A., Baral B., Debata P.R. (2022). Shifting of cell cycle arrest from the S-phase to G2/M phase and downregulation of EGFR expression by phytochemical combinations in HeLa cervical cancer cells. J. Biochem. Mol. Toxicol..

[106] Cortez A.J., Tudrej P., Kujawa K.A., Lisowska K.M. (2018). Advances in ovarian cancer therapy. Cancer Chemother. Pharmacol..

[107] Essa M.M., Bishir M., Bhat A., Chidambaram S.B., Al-Balushi B., Hamdan H., Govindarajan N., Freidland R.P., Qoronfleh M.W. (2023). Functional foods and their impact on health. J. Food Sci. Technol..

[108] Nandi S., Sikder R., Rapior S., Arnould S., Simal-Gandara J., Acharya K. (2024). A review for cancer treatment with mushroom metabolites through targeting mitochondrial signaling pathway: In vitro and in vivo evaluations, clinical studies and future prospects for mycomedicine. Fitoterapia.

[109] Islam S.M.R., Siddiqua T.J., Kabir Y. (2020). 20—Functional foods in cancer prevention and therapy: Recent epidemiological findings. Functional Foods in Cancer Prevention and Therapy.

[110] Yi L., Zongyuan Y., Cheng G., Lingyun Z., Guilian Y., Wei G. (2014). Quercetin enhances apoptotic effect of tumor necrosis factor-related apoptosis-inducing ligand (TRAIL) in ovarian cancer cells through reactive oxygen species (ROS) mediated CCAAT enhancer-binding protein homologous protein (CHOP)-death receptor 5 pathway. Cancer Sci..

[111] Ji H., Zhang Z., Chen C., Xu W., Liu T., Dong Y., Wang J., Wang H., Zhu X. (2024). The impact of quercetin and paclitaxel combination on ovarian cancer cells. iScience.

[112] Hashemzaei M., Delarami Far A., Yari A., Heravi R.E., Tabrizian K., Taghdisi S.M., Sadegh S.E., Tsarouhas K., Kouretas D., Tzanakakis G. (2017). Anticancer and apoptosis-inducing effects of quercetin in vitro and in vivo. Oncol. Rep..

[113] Dhanaraj T., Mohan M., Arunakaran J. (2021). Quercetin attenuates metastatic ability of human metastatic ovarian cancer cells via modulating multiple signaling molecules involved in cell survival, proliferation, migration and adhesion. Arch. Biochem. Biophys..

[114] Li N., Sun C., Zhou B., Xing H., Ma D., Chen G., Weng D. (2014). Low concentration of quercetin antagonizes the cytotoxic effects of anti-neoplastic drugs in ovarian cancer. PLoS ONE.

[115] Reyes-Farias M., Carrasco-Pozo C. (2019). The Anti-Cancer Effect of Quercetin: Molecular Implications in Cancer Metabolism. Int. J. Mol. Sci..

[116] Peeri N.C., Bertrand K.A., Na R., De Vivo I., Setiawan V.W., Seshan V.E., Alemany L., Chen Y., Clarke M.A., Clendenen T. (2025). Understanding risk factors for endometrial cancer in young women. JNCI J. Natl. Cancer Inst..

[117] Park S.L., Goodman M.T., Zhang Z.F., Kolonel L.N., Henderson B.E., Setiawan V.W. (2010). Body size, adult BMI gain and endometrial cancer risk: The multiethnic cohort. Int. J. Cancer.

[118] Kedzia M., Basta P., Czajkowski K., Gogacz M., Spaczynski R., Mroczkowska B., Stojko R., Szaflik T., Szubert M., Szyllo K. (2024). Guidelines of the Polish Society of Gynecologists and Obstetricians on the management of women with endometriosis. Ginekol. Pol..

[119] de Neufville Lucas A.R. (2014). Lifestyle Interventions for Endometrial Cancer Survivors: Feasibility and Efficacy of a Novel Mindfulness and Dietary Counseling Program. Ph.D. Thesis.

[120] Slighoua M., Amrati F.E., Chebaibi M., Mahdi I., Al Kamaly O., El Ouahdani K., Drioiche A., Saleh A., Bousta D. (2023). Quercetin and Ferulic Acid Elicit Estrogenic Activities In Vivo and In Silico. Molecules.

[121] Shi Y., Williamson G. (2016). Quercetin lowers plasma uric acid in pre-hyperuricaemic males: A randomised, double-blinded, placebo-controlled, cross-over trial. Br. J. Nutr..

[122] Lee J.-S., Cha Y.-J., Lee K.-H., Yim J.-E. (2016). Onion peel extract reduces the percentage of body fat in overweight and obese subjects: A 12-week, randomized, double-blind, placebo-controlled study. Nutr. Res. Pract..

[123] Brüll V., Burak C., Stoffel-Wagner B., Wolffram S., Nickenig G., Müller C., Langguth P., Alteheld B., Fimmers R., Naaf S. (2015). Effects of a quercetin-rich onion skin extract on 24 h ambulatory blood pressure and endothelial function in overweight-to-obese patients with (pre-)hypertension: A randomised double-blinded placebo-controlled cross-over trial. Br. J. Nutr..

[124] Li H., Li M., Fu J., Ao H., Wang W., Wang X. (2021). Enhancement of oral bioavailability of quercetin by metabolic inhibitory nanosuspensions compared to conventional nanosuspensions. Drug Deliv..

[125] Joseph A., Shanmughan P., Balakrishnan A., Maliakel B. (2022). Enhanced Bioavailability and Pharmacokinetics of a Natural Self-Emulsifying Reversible Hybrid-Hydrogel System of Quercetin: A Randomized Double-Blinded Comparative Crossover Study. ACS Omega.

[126] Van Zanden J.J., van der Woude H., Vaessen J., Usta M., Wortelboer H.M., Cnubben N.H., Rietjens I.M. (2007). The effect of quercetin phase II metabolism on its MRP1 and MRP2 inhibiting potential. Biochem. Pharmacol..

[127] Justino G.C., Santos M.R., Canário S., Borges C., Florêncio M.H., Mira L. (2004). Plasma quercetin metabolites: Structure-antioxidant activity relationships. Arch Biochem. Biophys.

[128] Day A.J., Mellon F., Barron D., Sarrazin G., Morgan M.R., Williamson G. (2001). Human metabolism of dietary flavonoids: Identification of plasma metabolites of quercetin. Free. Radic. Res..

[129] Dian L., Yu E., Chen X., Wen X., Zhang Z., Qin L., Wang Q., Li G., Wu C. (2014). Enhancing oral bioavailability of quercetin using novel soluplus polymeric micelles. Nanoscale Res. Lett..

[130] Singh A., Kureel A.K., Dutta P., Kumar S., Rai A.K. (2018). Curcumin loaded chitin-glucan quercetin conjugate: Synthesis, characterization, antioxidant, in vitro release study, and anticancer activity. Int. J. Biol. Macromol..

[131] Manzoor M.F., Hussain A., Sameen A., Sahar A., Khan S., Siddique R., Aadil R.M., Xu B. (2021). Novel extraction, rapid assessment and bioavailability improvement of quercetin: A review. Ultrason. Sonochem..

[132] Nishimuro H., Ohnishi H., Sato M., Ohnishi-Kameyama M., Matsunaga I., Naito S., Ippoushi K., Oike H., Nagata T., Akasaka H. (2015). Estimated daily intake and seasonal food sources of quercetin in Japan. Nutrients.

